# Ancient Mode of Treating Persons Bitten by Mad Dogs

**Published:** 1828-07

**Authors:** Frederick Adams

**Affiliations:** Surgeon.


THE LONDON
Medical and Physical Journal.

N° 353, VOL. LX.] JULY, 1828. [N° 25, *New Series.*

For many fortunate discoveries in medicine, and for the detection of numerous errors, the world is indebted to the rapid circulation of Monthly Journals; and there never existed any work, to which the Faculty, in Europe and America, were under deeper obligations, than to the *Medical and Physical Journal of London*, now forming a long, but an invaluable, series.—RUSH.

### ORIGINAL PAPERS,

AND

CASES OBTAINED FROM PUBLIC INSTITUTIONS AND OTHER AUTHENTIC SOURCES.

### HYDROPHOBIA.

### *Ancient Mode of Treating Persons bitten by Mad Dogs.*

By FREDERICK ADAMS, Esq. Surgeon.

EVERY remedial measure recently tried for the prevention and cure of hydrophobia having confessedly proved ineffectual, I beg to call the attention of the profession to a method of treatment which is said to have proved eminently successful in ancient times. I allude particularly to the internal use of the Veratrum Album, or White Hellebore, to which, however, as an auxiliary, was commonly joined the external application of the actual cautery to the wound. In order that the reader may be enabled to form an unprejudiced judgment of the testimony in favor of this plan of treatment, I shall now lay before him the opinions of some of the greatest authorities on ancient medicine, in their own words, literally translated.

" After the wounds have become cicatrised, the white hellebore is to be given for the complete removal of this affection; for, when this substance is given with cake to rabid dogs, they vomit, and are freed from the complaint."—AETIUS, Tr. ii. se. 2.

" The most effectual remedy which we know is the repeated use of hellebore."—PAULUS ÆGINETA, lib. v. c. 3.

" By far the most effectual cure for hydrophobia is the administration of hellebore."—ACTUARIUS, *De Meth. Med.* lib. vi. c. 11.

"Mad dogs are to be confined, and kept without food for one day. Let some hellebore be then mixed with their drink, and, after they have been purged by it, let them be fed with barley bread. In this manner you may likewise cure those who have been bitten by rabid dogs."—THEOMNESTUS AP. GEOPONICA, lib. xix. c. 3.

For the prevention of hydrophobia, "the most efficacious of all known remedies is a course of hellebore, which may be given with confidence once and again, and may be frequently repeated before the fortieth day, and even afterwards. Such is said to be the efficacy of this remedy, that certain persons who had began to feel the dread of water have been saved by taking the hellebore at the very commencement of the complaint. No one, however, who has been thoroughly affected with it has ever recovered."—DIOSCORIDES, lib. vi. c. 39.

CÆLIUS AURELIANUS, a timid and formal practitioner, of the sect called the Methodical, (whose practice in this disease is highly disapproved of by GALEN,) gives the following evidence in favor of the use of hellebore, although he himself condemns it.

"NIGER gave white hellebore for hydrophobia. EUDEMUS, after letting blood, gives hellebore on the second or third day, and applies cupping-glasses so as to raise blisters on the part. AGATINAS, in a work on Hellebore, orders hellebore in the commencement of the affection. Some direct a cataplasm of hellebore to be applied to the anus. Others introduce suppositories of hellebore."—*De Morbis Acut.* lib. iii. c. 16.

Such is the evidence in favor of the treatment of hydrophobia by hellebore; and, considering that it comprehends the testimony of at least thirteen centuries, it is surely deserving of some attention, after all our ephemeral plans of cure have fallen into disrepute. Hellebore, indeed, is a medicine of which most practitioners of the present day have little or no experience, and, considering the dreadful effects which ORIBASIUS describes as arising from the too liberal administration of it, there can be no question but that it ought to be given with extreme caution; yet, under proper regulations, there can be no doubt that it might be administered without danger, and, if any credit be due to the evidence of antiquity, with decided benefit, not only in this but in many other diseases. It is impossible to read the glowing eulogy which ARETÆUS bestows upon this medicine at the conclusion of his work, as it has come down to us, without being impressed with the conviction that hellebore must have deserved, in some degree at least, the confidence which the ancient physicians reposed in it.

The external application in which the ancients most trusted was the actual cautery; and in this they have been supported by the example of many eminent physicians of modern times. I may instance, in particular, Van Helmont, Stahl, Morgagni, and more recently, Maunoir, Marochetti, and Orfila, as distinguished abettors of this practice. None of them, however, seem to have acted up entirely to the principle upon which the ancients proceeded, or to have had any other object in view than to destroy the virus by the force of fire; whereas the ancients had it in view also to promote the discharge of it from the body, by keeping the wound open as an outlet to it. The nearest approach to the ancient method is that which is said to be pursued with great success at Breslau and Zurich, an account of which was published in Hecker's Litterarische Annalen for June 1825, and republished in the Edinburgh Medical and Surgical Journal for October 1825. The journalists seem in doubt whether the merit of priority be due to Breslau or Zurich; but the following extracts, while they confirm the strength of later evidence, will show that neither has any claim to originality.

"The part is to be drawn by a cupping glass;* and, if it is not nervous or muscular, the wound is to be burned. If it cannot be burned, it will not be improper to make it bleed. Then applications fit for burned parts are to be put upon the wound. When fire is not used, powerfully escharotic applications are to be had recourse to."—Celsus *de Medicina*, lib. v. c. 27.

"Having ascertained that the wound has been produced by the bite of a mad dog, you ought not, like the methodists, to heal up the sore in the common manner, but, on the contrary, you ought to enlarge it by cutting away the surrounding flesh to a considerable extent, and making it of a circular shape, so that it may be the longer of healing, and may remain open for the space of at least forty days,† that the poison of the dog may be discharged by it. Wherefore we are likewise in the practice of using heated cauteries for burning the part, and also other epispastic substances, so as not to allow the venom to lodge in it." Galen.

"The part which has been bitten by a mad dog is to be kept for a long time in a state of ulceration; and it is not to be permitted to cicatrise, in order that the virus may be discharged by it." —Scribonius Largus *de Comp. Med.*

"The most efficacious remedy for poisoned wounds is burning.

* This practice has been lately recommended by Dr. Barry.

† The following is a description of the Zurich plan of treatment: "Deep scarifications of the wound; besmearing it with Pulvis Lyttæ; application of a blister in the neighbourhood of the part; keeping up of suppuration, both in the blistered and wounded part, *during six weeks*, &c." Can any body endure all this torture?

It is to be particularly attended to that, when the eschars fall off, the lips of the wounds do not immediately coalesce; but the ulcers ought, if possible, to be kept open for a considerable time."—DIOSCORIDES, *u. s.*

AETIUS gives the following directions for the treatment of the wound:

"In the first place, the wound is to be enlarged by the scalpel; and the incisions are to be irritated so as to occasion a flow of blood from the part. The ulcer is afterwards to be burned with heated irons of a large size, and then we are to apply leek, or bread mixed with pounded salt, or onions, or garlic. And, when the eschars have fallen off, the ulcers are to be prevented from healing for forty or sixty days; or, if they have cicatrised, they are to be reopened." For this purpose he recommends various applications, and, among others, a composition, for which the following is a receipt:

℞. Salis Fossilis ʒ viij.; Chalcitidis ʒ xvj.; Squillæ ʒ xvj.; Rutæ Viridis ʒiv.; Æruginis Rasæ ʒ iv.; Marrabii Sem. ʒ j. M.

The chalcitis was a preparation of copper, resembling what is now called the sulphate; and this and the ærugo combined must have rendered the ointment a powerful escharotic, and consequently eminently calculated to fulfil the purpose for which it was intended.—See Tetr. ii. sec. 2.

PAULUS ÆGINETA gives the following formula for a composition intended to keep the ulcer open:

℞. Aceti Sextar. j. ℥xx.; Picis Purgat. ℔j.; Opoponacis Quadrant. ℥iij. M.

"Burning with iron is applied for the cure of diseases, but most particularly for the bite of a mad dog. Even after the persons bitten have become affected with the terror of water, they are speedily relieved by the application of the cautery to the wound." PLINY, *H. N.* lib. xxxiv. c. 44.

"By far the most efficacious remedy for poisoned wounds is burning; for, since the power of fire is greater than that of any other substance, it not only subdues the virus, and prevents it from spreading farther, but the part which is destroyed by it also contributes, in no small degree, to the cure, by remaining long in a state of ulceration. For it ought to be particularly attended to, that the parts be not allowed to coalesce sooner than proper."—ACTUARIUS, *De Meth. Med.* lib. vi. c. 11.

Regarding regimen, AETIUS prudently directs that the diet be neither too generous nor very spare, and intimates that he considers the latter extreme the more dangerous. In this respect his practice seems to have been founded upon very correct principles, since the absorbing powers of the veins are known to be in the direct ratio of their emptiness. This physiological doctrine, to the discovery of which MAGENDIE has lately laid claim, was well understood by the ancients, and is particularly inculcated by the Arabic medical writers. See AVERRHOES, *Colliget.* lib. vii. c. 1, and *Collectan.* § iii.—Upon this principle, I cannot but think that, although blood-letting be mentioned by some of the ancient writers, and is much depended upon by several modern surgeons, it ought to be entirely proscribed as a preventive of the disorder.

*Banchory ; May 4th,* 1828.

